# Emotional, mental health and physical symptom experience of patients hospitalized with COVID-19 up to 3 months post-hospitalization: A longitudinal study

**DOI:** 10.1111/jocn.16880

**Published:** 2023-09-12

**Authors:** Mary Fran Tracy, Sandy Hagstrom, Michelle Mathiason, Sarah Wente, Ruth Lindquist

**Affiliations:** 1 University of Minnesota School of Nursing, Minneapolis, Minnesota, USA; 2 M Health Fairview, Minneapolis, Minnesota, USA

**Keywords:** COVID-19, emotions, longitudinal studies, mental health, nursing, PASC, quality of life, SARS-CoV-2, self-report, symptoms

## Abstract

**Aims and objectives:**

To explore emotional, mental health and physical symptoms up to 3 months after discharge for adults hospitalized with COVID-19.

**Background:**

10%–30% of adults with COVID-19 experience physical and psychological symptoms 3 months or more following infection. Knowing symptoms can help direct early intervention.

**Design:**

A longitudinal descriptive design to study COVID-related symptoms 2 weeks, 6 weeks and 3 months after hospitalization.

**Methods:**

Sixty-six patients were recruited from a hospital system in Midwestern US (October 2020-May 2021). Participants self-reported demographics, hospital and post discharge symptoms, PROMIS measures (depression, anxiety, fatigue, cognitive function, satisfaction social roles, sleep disturbance) and Impact of Event Scale-Revised (IES-R). Hospital length of stay, comorbidities, lowest oxygen saturation, respiratory support and resources used were collected. Descriptive and nonparametric statistics described the sample and identified correlations between variables. The STROBE checklist was used.

**Results:**

Data from 1 (T1) and 3 months (T2) post discharge were analysed (*N* = 52). A majority were female, white and married; 96% experienced ≥1 COVID-related symptoms at T1; 85% at T2. Fatigue was most prevalent, followed by shortness of breath, muscle weakness and foggy thinking. More physical symptoms during hospitalization correlated positively with number of symptoms at T1 and T2; a majority stated these impacted their normal routine ‘somewhat’ or ‘a lot’. T1 depression highly correlated with all T2 PROMIS and IES-R scores and number of physical symptoms. More symptoms at T1 were associated with worse fatigue, lower cognitive function and lower satisfaction with social roles at T2.

**Conclusion:**

This study adds to the growing knowledge of mental, physical and emotional symptoms and relationships between these early after hospitalization with COVID-19.

**Relevance to clinical practice:**

Findings can help identify holistic nursing interventions to improve health and mitigate symptoms for people with long COVID.

**Patient or public contribution:**

Patients contributed via study participation.

## 1 ∣ INTRODUCTION

It is well noted that physical symptoms and psychological sequelae linger in many people after active infection with coronavirus disease-2019 (COVID-19) (Bellan et al., 2021; Lopez-Leon et al., 2021). Persons with COVID-19-related symptoms experienced for 3 months or more are often said to have ‘long COVID’, which has affected an estimated 16.3 million working age-Americans (Bach, 2022). Some COVID-19 sequelae are related to the disease itself, such as sudden and detrimental decompensation and severe, prolonged shortness of breath, which are known to create anxiety. Other sequelae may be related to the emotional toll of the illness. For those patients who are hospitalized with COVID-19, understanding the symptom experience and the resources needed and/or utilized by patients in the post discharge period is essential so that nurses can assess and intervene early, minimally for symptom management and ideally to promote full recovery and quality of life. Early intervention may have the potential to relieve symptoms, prevent ongoing or more severe sequelae and support optimal overall physical, emotional and mental health. This article reports findings from longitudinal research that followed patients hospitalized with COVID-19 for up to 3 months post discharge to explore their physical symptom and emotional and mental health experiences from a holistic perspective.

## 2 ∣ BACKGROUND

### 2.1 ∣ Post-acute sequelae of SARS-CoV-2 (PASC)

While a new and evolving concept at the time of this research, it has become clear that some patients have multi-system organ dysfunction and ongoing symptoms lasting well after the active infectious phase resolved (National Academies of Sciences, Engineering and Medicine, 2022a). This post-acute illness has been referred to as long COVID, post-COVID syndrome and post-acute sequelae of SARS-CoV-2 (PASC). Due to the evolving nature of COVID and postacute infection cases, the scientific community has not yet landed on one common definition for PASC. The U.S. Centers for Disease Prevention and Control (CDC) defines PASC as symptoms occurring four or more weeks in anyone post-COVID infection including those with mild or even asymptomatic cases, and estimates that PASC is experienced by one in five adult patients diagnosed with COVID-19 (CDC, 2021).

The World Health Organization (WHO) defines someone with PASC as meeting several criteria: having symptoms 3 months from the onset of COVID-19 symptoms that are either of new onset following recovery from COVID-19 or persisting from the initial illness, lasting for minimally 2 months, and including symptoms that cannot be explained by other diagnoses (WHO, 2021). As of August 2022, the WHO cites nearly 600 million people have had COVID-19 (WHO, n.d.). While Groff et al. (2021) reported in a systematic review that the median frequency of people infected with COVID-19 who develop PASC was approximately 55% in both high and low income countries, even a more conservative estimate, such as the 10%–30% cited by Bull-Otterson et al. (2022), would indicate that there may be hundreds of millions of people experiencing PASC. Despite this extraordinary number of people experiencing PASC worldwide, the global scientific community continues to have limited knowledge of the extent of the long-term disease course with the potential for symptoms to last weeks, months or years (NASEM, 2022b).

PASC is frequently a diagnosis arrived at from ruling out other potential diagnoses, in part because symptoms can be diverse and varied in severity, can sometimes be quite vague and can occur months after the initial COVID-19 infection; thus, providers may find it challenging to recognize PASC (McSpedon, 2022; NASEM, 2022b). Given these diagnostic difficulties, the challenges for patients to access appropriate care and resources is compounded (McSpedon, 2022). To optimize care and clinical patient outcomes, it is important for health professionals to learn more about patients' experiences, avoid dismissing complaints from COVID-19 survivors, and until definitive answers are known, utilize and treat patients using knowledge from other related diseases and symptoms (NASEM, 2022b).

### 2.2 ∣ Physical and psychological effects related to critical illness

Prior to COVID-19, much of our experience with negative psychological and physical sequelae in hospitalized patients has been in the critically ill population—particularly those who experience acute respiratory distress syndrome (ARDS) and/or sepsis requiring mechanical ventilation, diagnoses that appear to also be prevalent when patients become severely ill with COVID-19. In an early report of 119 patients hospitalized with COVID-19, 59% were diagnosed with sepsis, 54% with respiratory failure and 31% with ARDS (Zhou et al., 2020). Later reports have supported the findings that patients post-COVID can experience long-term respiratory, motor and cognitive impairment as well as post-traumatic stress symptoms and significant fatigue (Bailey et al., 2021; Bellan et al., 2021; Groff et al., 2021; Lopez-Leon et al., 2021; Weerahandi et al., 2021).

Post *Intensive Care Syndrome* (PICS) is a term used to describe the post discharge physical, mental health and cognitive dysfunction that persist well after discharge for intensive care unit (ICU) patients (Brown et al., 2019). Patients with PICS report issues with mental health access and financial difficulties (e.g. low rates of return to work) (Heydon et al., 2020). PASC could be viewed similarly to PCIS (Parker et al., 2021), though potentially reaching beyond the ICU walls to those hospitalized with COVID-19 on medical units, as well as those with mild COVID-19 cases not requiring hospitalization (NASEM, 2022b). While little has been published about the potential long-term sequelae (particularly psychological) of patients hospitalized on general medical units, it is not unreasonable to suppose that factors contributing to the psychological stress from being hospitalized in an ICU setting or with prolonged and strict physical and social isolation with a severe infectious disease may also occur in patients hospitalized with COVID-19 in a medical unit setting, including fear, nightmares, inability to communicate and guilt. In a study by Dziadzko et al. (2017), patients identified factors that mitigated PICS experiences including: family presence (just being there, physical contact, explaining to patient, reassurance), clergy, physical therapy/walking, nurse presence (holding hand, reassuring, encouragement, explaining, physical touch), physician attention and ability to communicate. These interventions are deeply embedded within the holistic care of nursing and could be purposefully utilized with people at risk for developing PASC.

### 2.3 ∣ Post-hospitalization follow up and services

Brown et al. (2019) have proposed four phases of critical illness: acute illness, hospital recovery, early post discharge recovery and late post discharge recovery; these authors suggest that full recovery is not only challenged by the resulting morbidity, but also from fragmented and inadequate care. Randomized controlled trials of interventions intended to mitigate these issues for ICU survivors with PICS have shown mixed results. Some healthcare systems have created ‘Aftercare and Recovery Clinics’; however, results of these efforts have been inconclusive, perhaps in part due to initiation of ‘visits more than three months after hospital discharge, well after a relevant window of vulnerability’ (Brown et al., 2019, p. 950).

The need for aftercare for those suffering from PASC is equally challenging and compelling due to their multifaceted needs, need for complex screening processes and presence of symptoms regardless of initial illness severity (Albu et al., 2021; Parker et al., 2021). Post-COVID Care Centers (PCCCs) have been initiated for COVID-19 survivors in 48 U.S. states as well as in Africa, Australia, Asia, Canada, Caribbean, Europe, India, Middle East and South America to date (Survivor Corps, 2022). It is unclear the timing of referrals to these centres, the extent to which issues beyond physical symptoms are addressed, and whether or how many PCCCs include nurses and nursing interventions to address the holistic needs of patients as nurses are not specifically identified as one of the disciplines to include in proposed PCCC Standard of Practice Guidelines (Survivor Corps, 2021).

Brown et al. (2019) posed future research to improve the care of PICS survivors by identifying services required, unmet needs and distinct groups that may have different needs and adverse outcomes. These same areas could be explored for survivors of COVID-19, regardless of whether they were hospitalized in the ICU or on a medical unit. While there has been a call to action to consider the long-term needs of large numbers of patients with COVID-19 post-ICU stay (Stam et al., 2020), there may be an even larger number of patients who were never admitted to an ICU but who may also have long-term sequelae from COVID-19 and longer than usual hospital stays.

This study is an imperative first step to describe patients' experiences in order for health care providers to identify interventions that will ameliorate and ideally prevent development of long-term consequences of COVID-19. The purpose of this paper is to report on the emotional, mental health and physical symptoms of patients with COVID-19 in the early post discharge recovery period (i.e. up to 3 months post discharge) and the resources this sample used or would have liked to have had available during that time frame in order to provide a foundation for nursing interventions in this population.

## 3 ∣ METHODS

### 3.1 ∣ Design

A longitudinal descriptive design was used to evaluate patient self-reported experience of COVID-related symptoms at 2 weeks, 6 weeks and 3 months after hospitalization for treatment of the primary diagnosis of COVID-19. The Strengthening the Reporting of Observational Studies in Epidemiology (STROBE) Statement: Guidelines for Reporting Observational Studies checklist was used (File S1).

### 3.2 ∣ Sample and setting

Patients who had been hospitalized and discharged from one of 11 hospitals in an academic-affiliated healthcare system in the Midwest United States were eligible to participate. Inclusion criteria included: ≥18 years of age, able to speak and read English, hospitalized on a medical and/or intensive care unit in this healthcare system, and had access to either an electronic device or phone for communication and survey participation.

Due to pandemic challenges, there was a multi-pronged approach to participant recruitment.

- Eligible participants were identified through the health system's research administration office that reviewed discharges daily for patients whose primary reason for admission was COVID-19. These patients received notification via their secure electronic health record (EHR) portal and were provided a link to learn more about the study and directly complete the initial survey. If they indicated interest in the study but did not proceed to complete the survey, a member of the research team contacted them by phone to confirm interest and send them a link via their email or to offer assistance to complete surveys over the phone.
- Additional participants were identified through the academic Clinical and Translational Science Institute's COVID-19 Registry. A research flyer was mailed to eligible participants' home addresses describing the study and providing a link to the survey and an email/phone number to contact the research team with questions. A member of the team contacted those who reached out and either sent them the survey link via email or offered to assist in completing surveys over the phone.

Participants received a gift card of up to $50 after study completion as a thank you for participating in the research; monetary value was based on the number of surveys completed over the three-month post discharge period.

### 3.3 ∣ Data collection and measures

Enrolled participants were asked to complete electronic surveys at three time points through REDCap, a secure web application for building and managing online surveys (Harris et al., 2009). Targeted time frames for participants to complete surveys were 2 weeks post hospital discharge, 6 weeks post discharge and 3 months post discharge. Four participants completed the surveys over the phone with a researcher at the participants' request while the remainder completed the surveys electronically. In addition, information was gathered from the EHR related to participants' COVID-19 hospitalizations (e.g. medical/ICU unit(s), admission/discharge dates, discharge disposition, comorbidities at time of admission, levels of respiratory support, lowest oxygen saturation and presence of notes from ‘supportive’ services including chaplain, social work and therapies).

Self-reported demographics collected at the time of first survey completion included age, sex, race/ethnicity, marital status, household members, work status and number of hospitalizations in the previous 2 years. Hospital-related information self-reported in the first survey included symptoms they experienced in the hospital. They were also asked to identify up to five things that were most helpful to them to alleviate stress while in the hospital and up to five things that would have helped them with stress but which were not available or they could not do. Post discharge information collected at each time point included resources accessed or desired to access (e.g. primary care provider and social services), COVID-related symptoms in the 7 days preceding survey completion and impact of symptoms on their daily routine.

Standardized measures completed at each time point included the Impact of Events Scale-Revised (IES-R) and the following 8-item adult short form Patient Reported Outcome Measurement Information System (PROMIS) measures (English versions): Anxiety 8a, v1.0; Depression 8a, v1.0; Fatigue 8a, v1.0; Cognitive Function 8a, v2.0; Sleep Disturbance 8a, v1.0; and Satisfaction with Participation in Social Roles and Activities 8a, v2.0. PROMIS measures have been extensively studied and are available for use through the National Institutes of Health; short form versions have similar reliability and precision as the longer versions (HealthMeasures, 2020). All measures ask respondents to report perceived experience in the 7 days preceding survey completion using Likert-type responses such as never/not at all to always/very often. PROMIS measure T-score metrics have a mean of 50 based on an established general population which allows for normative score interpretation; ‘higher T-scores indicate more of the concept being measured. For measures of function, a higher score is better health (e.g. physical function); for measures of symptoms, a higher score is worse health (e.g. fatigue)’ (Hanmer et al., 2020, p. 5). T-scores from 55 to 60 indicate mild dysfunction, 60–70 moderate and >70 severe dysfunction on the depression, anxiety, fatigue and sleep measures. Cognitive function T scores of 40–45 are considered mild dysfunction, 30–40 moderate and <30 severe dysfunction. On the satisfaction with social roles and activities measure, T-scores between 30 and 40 are low and a score < 30 indicates very low satisfaction.

The Impact of Event Scale-Revised (IES-R) is a 22-item self-report measure developed and revised to assess a person's response to a specific trauma (COVID-19 hospitalization for these participants) (Weiss & Marmar, 1997) including core concepts of post-traumatic distress syndrome (PTSD): intrusion, avoidance and hyperarousal. It has been shown to be psychometrically sound in multiple populations and to discriminate between those who have experienced trauma and the general population. The tool can be used as a screening tool (rather than diagnostic) for PTSD. While this tool had not been specifically used in research with COVID-19 patients at the time of this study, it has demonstrated high sensitivity (80%–100%) and specificity (85%–91%) in survivors of acute lung injury (Bienvenu et al., 2013). Item responses are on a Likert-type scale ranging from not at all (0) to extremely (4); therefore, the total score can range from 0 to 88. Total scores from 24 to 32 raise a clinical concern that persons are experiencing some PTSD symptoms (Asukai et al., 2002), while scores of 33–38 can be seen as a cut-off for a probable diagnosis of PTSD (Creamer et al., 2003). Scores 39 or higher raise concern for physical impact for the person believed to indicate the potential to suppress immune system functioning up to 10years after an impact event (Kawamura et al., 2001).

### 3.4 ∣ Analysis

Due to the challenges of recruiting participants during a pandemic, as noted we utilized multiple methods for recruitment resulting in a wider range of survey completion times (both earlier and later) than originally planned in study design. For example, patients who received notice of the opportunity to participate via their EHR portal had immediate access to the study consent and initial survey for completion so timing varied based on when they opened their EHR and saw the study information. Since some survey questions requested reporting of symptoms/feelings experienced in the past 7 days, participants who completed their first survey within 7 days of discharge could report overlap of symptoms experienced in the hospital and immediate post discharge; these surveys were excluded from analysis for this report. In addition, there was time variation for participants who received information about the study via postal mailing based on when the letters were sent and opened. Therefore, we analysed data from participants who completed at least two surveys—one at approximately 4–6 weeks after hospital discharge (T1) and one at approximately 2–3 months (T2). For each participant, we chose the survey completed closest to 30 days for T1 (average 40.7 days post discharge, range 28–59 days) and the survey completed closest to 90 days for T2 (average 79.9 days post discharge, range 69–96 days).

Data analyses were performed using SAS statistical software. Descriptive statistics were used to describe the sample and PROMIS measure scores, physical symptom experiences. McNemar tests, nonparametric analyses, were used to test for changes in proportions. PROMIS R-scores were calculated by summing raw scores for each scale and then transformed to T-scores. IES-R is reported as a total score.

### 3.5 ∣ Ethical considerations

The research protocol was reviewed and approved by the university institutional review board (IRB) and by the health system's research administration and Nursing Research Proposal Review Committee.

## 4 ∣ RESULTS

**Figure 1.**
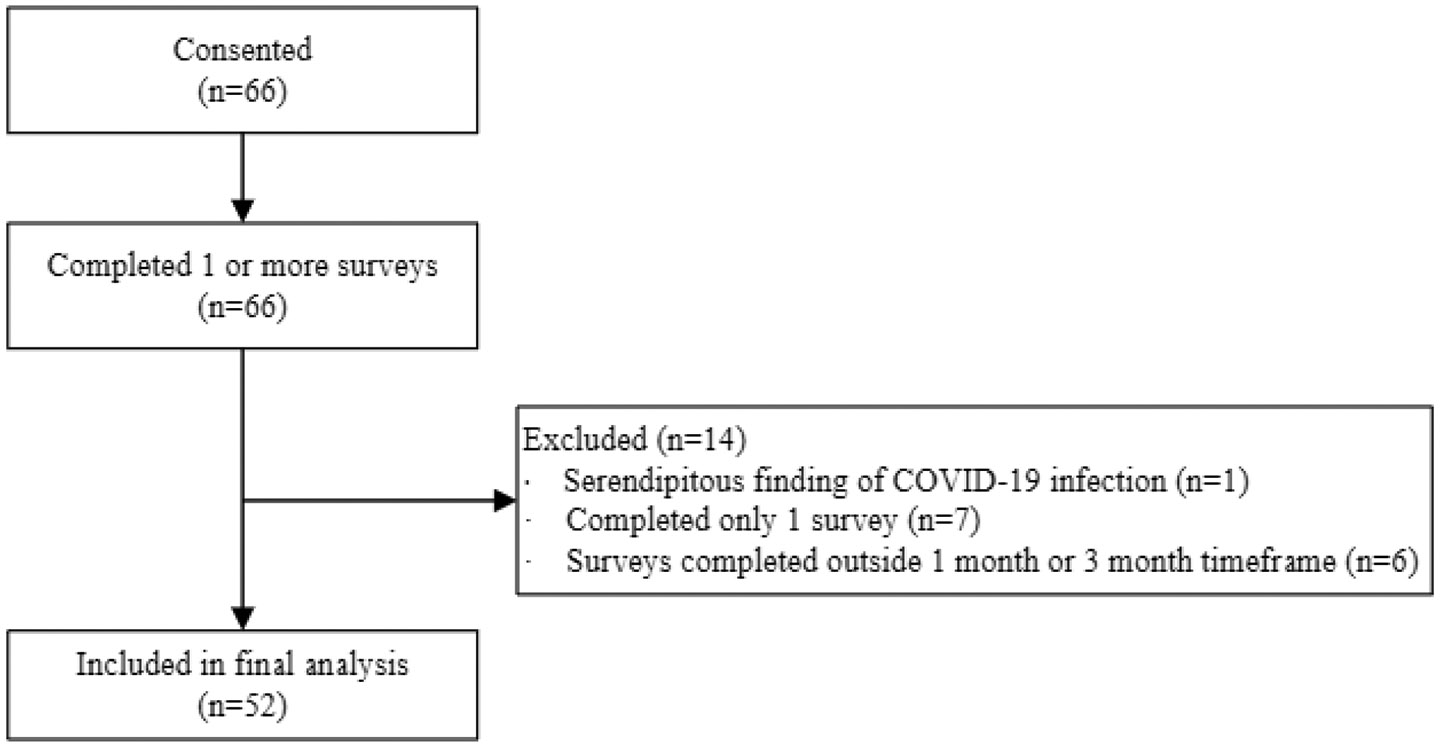
Flow diagram.

A total of 66 participants consented and completed at least one survey between October 2020 through May 2021. It was discovered that one participant tested positive for COVID-19 while hospitalized for a separate condition; because the positive COVID finding was serendipitous and the patient did not experience COVID-19 symptoms, this person was not eligible for participation and data were removed from analysis. Six participants completed two or three surveys but not in the appropriate post discharge timeframe. Seven participants only completed a single survey. These participants' data are not included in the final data analysis. This resulted in the final sample of 52 participants (Figure 1).

A majority of the sample were female (58%), white non-Hispanic (94%), married (71%) and employed prior to their COVID-19 illness (63%). The mean age was 57.9 years (SD 14.0; range 19–81), and 96% of participants had comorbidities identified at the time of admission. The average number of systems within which each participant had one or more comorbidities identified was 3.5 (SD 1.9); for some this included more than one comorbidity per body system (e.g. if the same participant had hypertension and a myocardial infarction, it counted singly as having the presence of a cardiac system comorbidity). One-fourth had been hospitalized at least one time in the 2 years before their admission for COVID-19 (Table 1).

### 4.1 ∣ Hospital experience

The median hospital length of stay (LOS) was 4.5 days (IQR 3–7). Six (12%) participants were admitted to an ICU during their hospital stay. Symptoms most commonly experienced in the hospital were fatigue (90%), respiratory (90%) and neurological (88%), though participants experienced multiple symptoms across body system categories; the mean number of symptoms reported per participant was 10.5 (SD 5.0) (Table 2). No significant correlations were found between any of the most common prior health conditions (i.e. cardiac, respiratory, endocrine and mental health) and number of symptoms in the hospital; similarly, total number of comorbidities per participant was not related to number of symptoms (Table 3). The highest level of respiratory support for each participant ranged from none (room air) to endotracheal intubation, with the majority requiring nasal cannula or oxygen mask (60%); the lowest oxygen saturation (SpO2) recorded during hospitalization was between 80% and 89% for 73% of participants (Table 1).

### 4.2 ∣ Physical symptom experience at T1 and T2

In the 7 days prior to T1 survey completion, 96% of participants experienced at least one COVID-related physical symptom, with mean number of symptoms 8.2 (SD 4.1; range 1–18); 85% still reported at least one physical symptom at T2 with average number of symptoms 8.0 (SD 4.0; range 2–17; Table 2). Number of symptoms in the hospital was correlated with number of symptoms at both T1 [*r*(50)=.50, *p*<.001] and T2 [*r*(50)=.41, *p*=.002] while LOS correlated with number of symptoms reported at T2 [*r*(50)=.30, *p*=.031; Table 3]. Fatigue continued to be the most common symptom reported at both T1 and T2 (87% and 77% respectively), followed by shortness of breath, muscle weakness, foggy thinking and aches. Nine participants reported new onset of numbness at T2. More than half of respondents (67% at T1, 54% at T2) reported that symptoms had continued to impact their ability to carry out their normal routine either ‘somewhat’ or ‘a lot’. Quality of life was reported as worse than before having COVID-19 by 69% of participants at T1 and 46% at T2. Comparison of responses to the quality of life question at T1 and T2 revealed no change for the majority (67%), improvement for 25%, worsening for 6% and large improvement (from worse at T1 to better than pre-COVID-19 at T2) for one person. Of those employed prior to contracting COVID-19 (*n*=35), 74% had returned to work at T1 and 86% at T2 (Table 2).

### 4.3 ∣ PROMIS measures and IES-R

Data from PROMIS measures indicated that on average participants had mild fatigue at T1 (mean=55.2; SD=10.2), though mean scores trended towards normal at T2 (mean=51.6; SD =10.6). The group mean also showed low average satisfaction with social roles and activities at T1 (mean=43.6, SD=8.6), trending towards normal limits at T2 (mean=48.1, SD 9.1). The means for all other PROMIS measures were within normal limits at both T1 and T2, and the mean IES-R total scores (13.5 at T1 and 11.0 at T2) demonstrated that on average participants were below the cut-off score that would raise clinical concerns for PTSD, although scores ranged widely (0–67 at T1, 0–66 at T2; Table 4).

**Figure 2.**
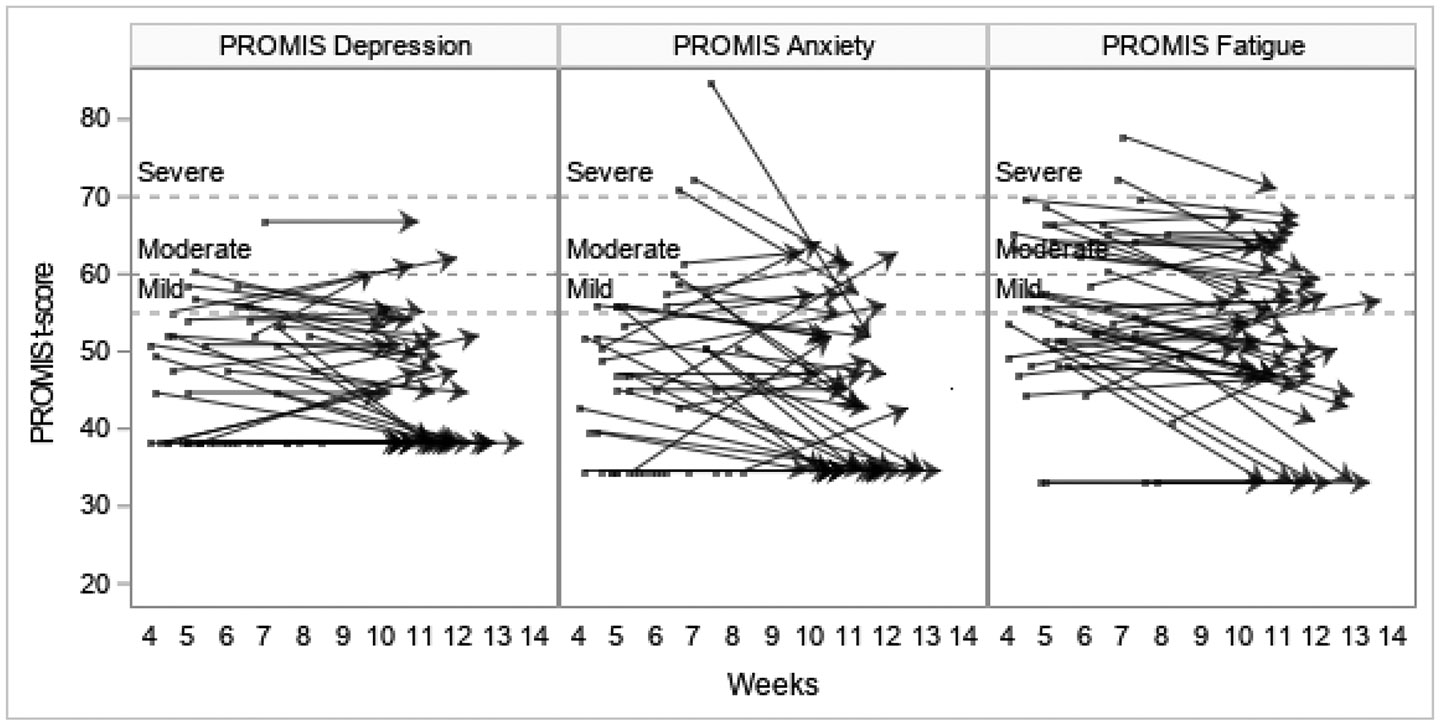
Individual participants’ PROMIS depression, anxiety, and fatigue T-scores showing change between T1 and T2. Score between 55-60 indicates mild dysfunction; 60-70 moderate dysfunction; > 70 severe dysfunction.

**Figure 3.**
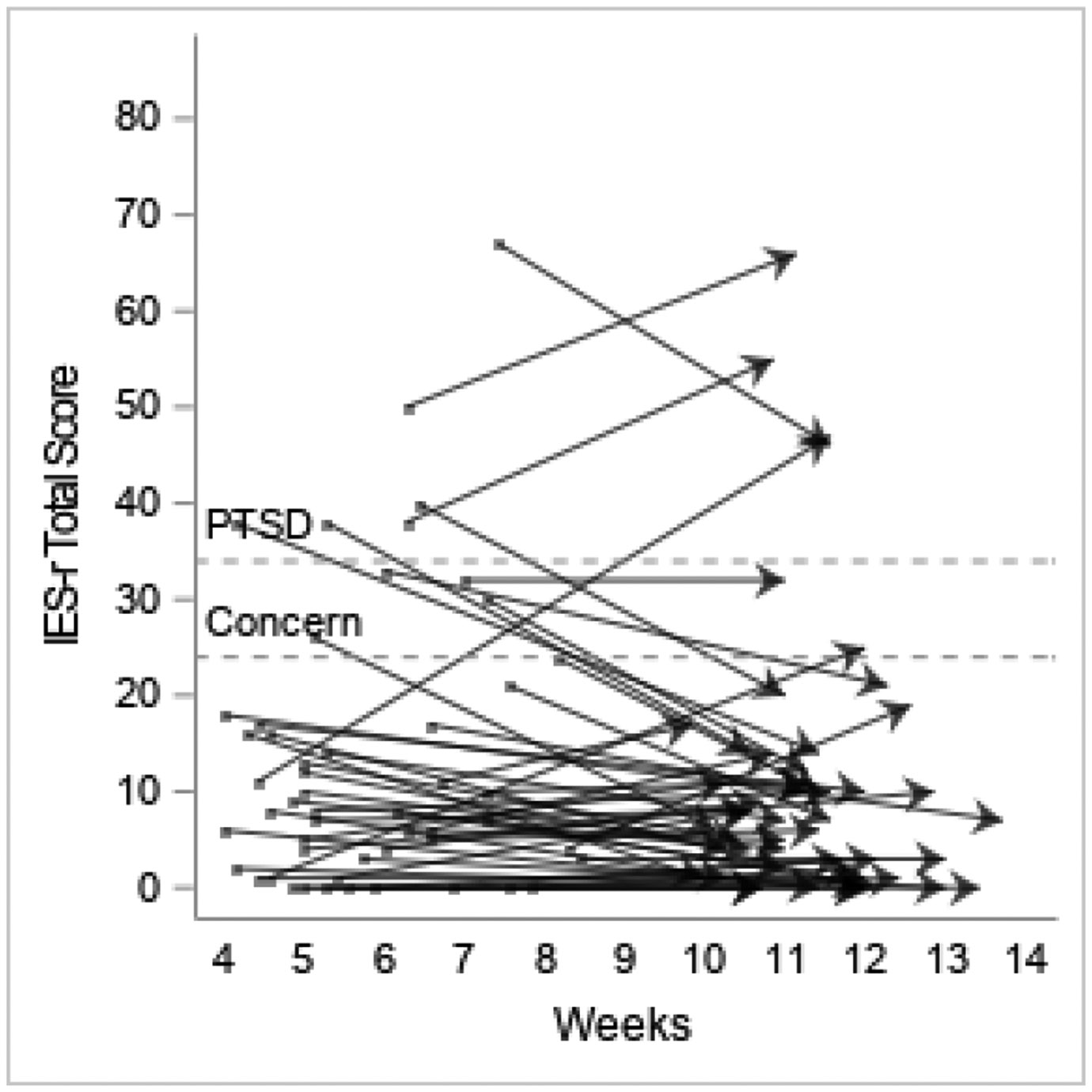
Individual participants’ Impact of Events Scale-Revised scores showing change between T1 and T2. Score between 24-32 indicates clinical concern for post-traumatic stress disorder (PTSD); >32 indicates probable PTSD diagnosis.

**Figure 4.**
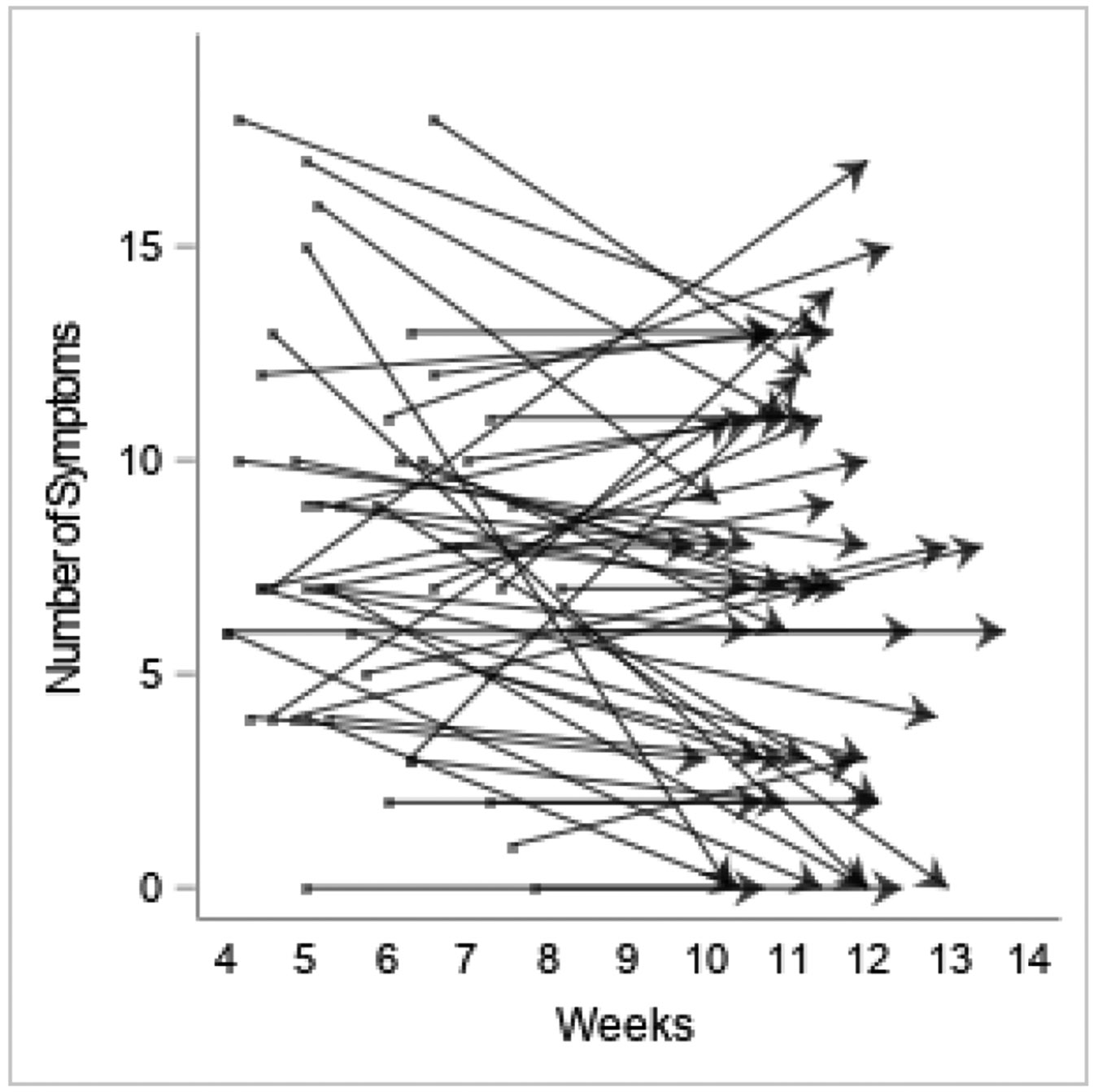
Individual participants’ number of self-reported symptoms showing change between T1 and T2.

Individual trajectories demonstrated various patterns, with the majority having normal scores on all measures at both T1 and T2 (Figures 2-4). However, there was a wide range in individual scores on each of the measures (Table 4). Some participants with scores indicating dysfunction on a particular measure at T1 showed improvement at T2, while others remained the same, and some participants whose scores were within normal limits at T1 indicated dysfunction at T2. Similar patterns were seen in number of symptoms experienced.

Moderate to strong correlations were seen between most of the standardized measures and number of symptoms within both T1 and T2 (e.g. T1 fatigue correlated with each T1 PROMIS measure and and IES-R) (Table 4). While significant correlations were not found between some T1 variables, at T2 the relationships between all PROMIS measures, IES-R and number of symptoms were significant with the exception of depression and sleep disturbance. No correlation was found between the number of prior comorbidities per participant and any of these variables (Table 3).

#### 4.3.1 ∣ Correlations among measures across time points

When comparing the most common prior health conditions to PROMIS, IES-R and number of symptoms at T1, depression was found to be positively associated with respiratory [*r*(49)=.31, *p*=.029] and mental health condition [*r*(49)=.35, *p*=.013], and negatively associated with cardiac condition [*r*(49)=−.30, *p*=.035]. Another significant correlation was between prior mental health condition and deficits in cognitive function [*r*(50)=−.30, *p*=.029]. However, at T2 no significant correlations were found between common prior health conditions and any PROMIS measure, IES-R or number of symptoms (Table 3).

Number of symptoms in the hospital correlated with patient-perceived cognitive deficits at both T1 [*r*(50)=−.60, *p*<.001] and T2 [*r*(50)=−.54, *p*<.001]. Associations were also found at both T1 and T2 between number of hospital symptoms and fatigue [*r*(50)=.39, *p*=.004 at T1; *r*(50)=.41, *p*=.002 at T2] and IES-R [*r*(50)=.28, *p*=.047 at T1; *r*(50)=.27, *p*=.050 at T2]. T2 anxiety correlated significantly with number of hospital symptoms [*r*(50)=.38, *p*=.006; Table 3].

Analysis of PROMIS and IES-R measures across T1 and T2 showed significant correlations within measures (e.g. depression at T1 correlated with depression at T2). Individual T1 measures also correlated with most T2 measures and number of symptoms with a few exceptions (Table 3). T1 depression was highly correlated with all T2 PROMIS measures, IES-R and number of symptoms. Higher number of symptoms at T1 was associated with T2 variables including higher fatigue [*r*(50)=.53, *p*<.001], lower cognitive function [*r*(50)=−.50, *p*<.001] and lower satisfaction with social roles and activities [*r*(50)=−.34, *p*=.015].

### 4.4 ∣ Resources used after hospitalization

At T1, 94% of participants reported accessing at least one resource since hospital discharge, with primary care provider as the most common (90%) (either virtual/phone visit or in-person visit or a combination) followed by pharmacist (52%) and specialist physician/ clinic (44%). There were a few reports of participants feeling a need to use a resource but having challenges in meeting that need. The most frequently used new medical equipment since hospital discharge was oxygen (27%), including one who reported use of oxygen around the clock instead of only at night (Table 5).

## 5 ∣ DISCUSSION

### 5.1 ∣ Sample demographics, characteristics and physical symptoms

This was a homogeneous and relatively young sample. Essentially, all participants had comorbidities prior to being hospitalized for COVID-19, though the majority had not been hospitalized in the 2 years prior to contracting COVID-19. The most frequent comorbidities noted from the EHR in this sample were those that have been reported to be risk factors for requiring hospitalization with COVID-19 (i.e. cardiac, respiratory and endocrine) (Bergman et al., 2021; Hamdan et al., 2021). Some of these comorbidities were directly related to the symptom measures we used post-hospitalization; for example, 37% of participants had baseline mental health diagnoses listed in their EHR (e.g. anxiety and depression). Symptoms from COVID-19 requiring hospitalization may have been similar to or an exacerbation of those symptoms related to pre-existing conditions; therefore, it can be difficult to distinguish what symptoms were solely related to the COVID-19 infection in the self-report measures post discharge. The median LOS was relatively short at 4.5 days; there were only a few patients who had no comorbidities, did not need oxygen and had short LOS. The longer the LOS, the more symptoms participants experienced at T2. Neither number nor type of comorbidities or age were associated with LOS.

Participants reported experiencing a myriad of symptoms in the hospital (mean 10.5 symptoms/participant); many of these symptoms continued post discharge, which is similar to other reported studies (Chen et al., 2022; Yoo et al., 2022). The more symptoms experienced in the hospital, the more symptoms were reported at T1 and T2. A large majority of participants appear to have met the definition of experiencing PASC at both T1 and T2 (96% and 85%, respectively). This is a much larger percent of people with ongoing symptoms than reported by Bull-Otterson et al. (2022), but similar to a meta-analysis (Lopez-Leon et al., 2021) where 80% of those patients reported long-term symptoms and a scoping review of studies of patients in rehabilitation by Wasilewski et al. (2022) which stated that of people who had symptoms at COVID-19 onset, 52% continued to have symptoms at 4weeks. The high number of participants who reported ongoing symptoms may be due to the fact that this sample came exclusively from those who were ill enough to require hospitalization and solely based on self-report. We were not able to determine whether their primary care providers had ruled out alternate causes other than COVID-19 for their reported symptoms.

While respondents reported that symptoms were generally decreasing from T1 and T2, there were some participants who still had significant numbers or extent of symptoms that were impacting their daily routine and quality of life. In addition, some participants reported new symptoms at T2 that they had not been experiencing at T1. It is clinically important to note that there were not statistically significant improvements for some symptom categories at T1 and T2. For instance, while the number of any symptoms and overall neurological symptoms did show a statistically significant improvement between T1 and T2 (*p*=.014 and *p*=.013, respectively), the majority of participants still had symptoms in each category except gastrointestinal. For example, 69% had lung symptoms at T1 and 56% still had them at T2; foggy thinking, memory, headaches and fatigue symptoms also were not significantly improving at T2. Participants who are still experiencing these symptoms at T2 are those defined as having ‘long COVID’ (PASC) by the CDC (2021) and the WHO (2021).

### 5.2 ∣ PROMIS and IES-R measures

Fatigue was the only PROMIS measure symptom whose mean deviated from the general population T scores at T1, showing mild dysfunction; fatigue trended towards the general T score at T2. The anxiety score had the highest maximum and the largest variation, which is illustrated in the individual trajectories demonstrating that participants with severe anxiety at T1 decreased to moderate, mild or normal at T2 while a number of individuals had mild or moderate anxiety at both T1 and T2, and some who were normal at T1 had moderate anxiety at T2 (Figure 2). Findings were similar for the IES-R responses where the mean did not indicate PTSD symptoms, but the large standard deviation (14.8) and range (0–67) indicate that some participants likely had PTSD related to the experience of being hospitalized with COVID-19; this persisted from T1 to T2 for some and was a new finding at T2 for others (Figure 3). The numbers of participants with scores indicating some dysfunction are not inconsequential and demonstrate the need for healthcare providers to minimally identify and continue to monitor patients who may be struggling with these issues and intervene with resources as necessary.

### 5.3 ∣ Relationships between and within health domain measures

There were statistically and clinically significant relationships noted in this study between and within the physical, emotional and mental health domains. For example, the more physical symptoms participants reported at T1, the lower both cognitive function and satisfaction at T2 as well as higher score on the fatigue PROMIS measure. Depression scores at T1 were highly correlated with all T2 PROMIS and IES-R scores; similarly, T1 anxiety was also correlated with all T2 variables, though not as strongly as T1 depression. T1 depression was also correlated with the number of physical symptoms at T2, though this correlation was slightly lower. The correlation of depression and anxiety with adverse outcome measures underscores the importance of early identification and treatment of these conditions in an effort to reduce symptom burden in recovery and improve quality of life. In addition, the worse the score on any one measure (PROMIS or IES-R) at T1, the worse the score on almost all other measures at both T1 and T2.

### 5.4 ∣ Use of resources

The majority of participants (90%) post discharge reported at least one visit with their primary care provider at T1 which is to be expected; in addition, 52% used the services of a pharmacist, 44% a specialist physician; few reported use of other resources such as the emergency department, therapies (occupational, physical, speech) or counsellors. Even fewer used any of these resources between T1 and T2, though many were still experiencing symptoms. This could align with the experience of patients who have historically experienced long-lasting effects from ICU admission where resources are not available or taken advantage of despite struggles with post-ICU syndrome (Brown et al., 2019). It is worth noting that this study occurred early in the pandemic when definitive treatments and follow-up resources were still being researched and developed, few COVID-19 follow-up clinics had been established, and in person access to resources was limited due to recommendations to avoid contact whenever possible.

### 5.5 ∣ Limitations and strengths

There were several limitations to this research study. This was a longitudinal design with a small demographically homogeneous sample of patients who were hospitalized with the SARS-CoV-2 virus; therefore, generalizability is limited. Data analyses revealed associations, not causal relationships; therefore, findings should be cautiously interpreted. The majority of information was self-reported, which does represent important individual reality and perspective. However, it was not feasible to have corresponding clinician assessments to confirm symptoms or more rigorously quantify or diagnose conditions such as clinical depression. Because of challenges identifying and recruiting potential participants, there was variation in the time frame in which participants completed the surveys in relation to their hospital discharge. Thus, time was an uncontrolled factor that may have affected accuracy of recall and reporting potentially leading to time biases.

One of the original aims of the study was to compare patients who were admitted to the ICU vs. those who were only ever on a medical unit. However, since we were able to recruit only a few ICU participants (likely due to the high mortality rates at this stage of the pandemic), we had to abandon this original study aim. We do recognize that due to the high census of most hospitals at this time, symptoms for a patient hospitalized on a medical unit with COVID-19 may have been very similar to either patients in the ICU or patients at home with COVID-19 simply because there were no hospital beds (either in ICUs or medical units) available.

A strength of this study is the broad assessment of symptoms explored (physical, emotional and mental health symptoms) in an effort to establish a holistic view of the experiences of these participants hospitalized with COVID-19. Tools were chosen (i.e. symptom checklists, PROMIS and IES-R) for their ease of use and potential use as routine screening tools during post discharge follow-up visits to identify ongoing concerns or patient trajectories. We believe this is one of the few studies conducted by nurses offering a nursing perspective of the hospital experience and recovery trajectory of patients hospitalized with COVID-19.

## 6 ∣ CONCLUSION

Even when using conservative estimates, hundreds of millions of people globally are or will experience PASC, hindering their full recovery from COVID-19 and their ability to maintain optimal quality of life. Findings from this study reveal relationships from a holistic nursing perspective between the physical symptom and emotional and mental health experience domains. These findings add to the growing body of evidence for these distressing sequelae and provide ideas for further research to deepen our knowledge and explore nursing interventions that are the most likely to be helpful for this population. Close follow-up with patients in the 1–3 months post-hospitalization for COVID-19 and use of short standardized PROMIS measures can assist nurses to identify PASC and provide early interventions to address the myriad of symptoms people with PASC can experience.

## 7 ∣ RELEVANCE TO CLINICAL PRACTICE

Information discovered from this research has the potential to impact how nurses care for COVID-19 patients. The tools used in this study were short, easy to use and widely available, making them easily incorporated into nursing routine assessments for COVID-19 patients. While the study was not designed to determine predictions or underlying causes of symptoms in these health domains, it does provide rationale for nurses to carefully explore how people are functioning physically, emotionally and mentally after being hospitalized for COVID-19 at multiple time points after discharge. Based on these data, we cannot assume that symptoms will resolve for all patients by 3 months post discharge, and in fact, they may be developing new symptoms that were not previously experienced.

Fatigue was the most common and persistent symptom experienced by participants over time (T1 and T2). Plans of care and structural hurdles to follow-up clinic visits ought to be adjusted to accommodate this fact. Goal setting for return to work and resumption of activities of daily living and other role responsibilities should be adjusted accordingly, also taking time to validate with patients and families the frustration and emotional, mental health and financial impact of not being able to return quickly to previous activity levels. Supports ought to be put in place for times and places to rest as needed and medical therapies sought to address the fatigue so commonly experienced.

Correlations between measures in this study indicate that minimally, if patients score high in PROMIS measures for depression, anxiety, or fatigue or have higher numbers of symptoms at T1, nurses should consider continuing to closely follow these patients as it is likely those symptoms may persist to T2 and/or they may develop new symptoms. This may be true regardless of their pre-existing comorbidities; for instance, while 37% of patients had a pre-existing diagnosis of a mental health condition, there were some without a diagnosed mental health pre-existing comorbidity who were also experiencing higher depression and anxiety scores. Consideration should also be given to the potential for PTSD related to the experience of having COVID-19, especially for patients who required hospitalization and/or continue to have distressing symptoms or high scores on PROMIS measures.

In addition, it may be useful for nurses to utilize short form PROMIS measures such as for fatigue vs. relying solely on patient anecdotal comments of fatigue; the measure can ‘quantify’ the extent of the symptom and allow for more precise tracking of improvements/worsening over time. Use of these instruments may also allow clinician recognition of the extent of symptoms, rather than regarding them as an expected consequence of routine recovery and dismissing complaints due to lack of COVID-19 recovery knowledge on the part of clinicians (NASEM, 2022b).

Patients with greater numbers of symptoms while hospitalized had more symptoms and health challenges at T1 and T2. Further, those with longer lengths of stay were also more impaired at T2. Perhaps, such patients warrant closer or more intensive follow-up and monitoring post discharge. Likewise, foggy thinking, memory impairments, headaches and fatigue reported at T1 were not significantly improved at T2. Over two thirds of patients at T1 and nearly half of patients at T2 reported that their quality of life was diminished as compared to baseline, prior to COVID-19. More work is needed to determine intervention strategies to foster patient improvement over time, or to provide support networks as patients and their families strive to recover or to reach a new normal and to return to the quality of life enjoyed prior to their COVID-19 hospitalization.

We hope our findings provide guidance for future research in many areas that can ultimately improve nursing practice; identifying not only the recovery trajectory of COVID-19 patients, but also strategies to monitor and optimize recovery. Practicing nurses can work to identify and implement resources that improve outcomes before and after hospital discharge; similarities and/or differences between people hospitalized with COVID-19 vs. those not hospitalized; and interventions to reduce the risk of developing longer-term emotional, mental health and physical disturbances from the impact of a sudden, unanticipated infectious disease. Researchers could determine the efficacy of those efforts. Likewise, researchers could work to measure longer-term outcomes beyond the three-month measurements in this study.

Wasilewski et al. (2022) emphasize that there needs to be more focus on the psychosocial aspects of recovery from a COVID-19 illness. This is an area that nurses can excel in addressing since it is well-aligned with our perspective of listening to patients describe their lived experiences, facilitating symptom management and providing holistic care (Pinto et al., 2021). As noted, we must ‘recognize that surviving serious illness is not the same as recovering; public understanding is growing that quality of life is also an important outcome’ (NASEM, 2022a, p. 4).

## Supplementary Material

Strobe Checklist

Additional supporting information can be found online in the Supporting Information section at the end of this article.

## Figures and Tables

**Table 1:** Participant Characteristics (N=52)

Characteristic	
Age in years, mean (SD)	57.9 (14.0)
Sex	
Female	30 (58%)
Male	22 (42%)
Ethnicity and race	
White (non-Hispanic)	49 (94%)
Hispanic or Latino	2 (4%)
Asian	1 (2%)
Marital status	
Married	37 (71%)
Divorced	5 (10%)
Widowed	4 (8%)
Never married	3 (6%)
Long term partner	3 (6%)
Work status prior to hospitalization	
Outside employment	28 (54%)
Retired	16 (31%)
Self-employed	5 (10%)
Unemployed, not due to COVID-19	2 (4%)
Work in home caring for family	1 (2%)
Number of comorbid conditions identified at time of admission	
Mean (SD, range)	3.5 (1.9, 0-8)
Comorbid conditions identified at time of admission	
Cardiac	26 (50%)
Lipid-related	25 (48%)
Respiratory	21 (40%)
Endocrine	21 (40%)
Mental health	19 (37%)
Obesity	16 (31%)
Gastrointestinal	13 (25%)
Neurological	8 (15%)
Renal	8 (15%)
Hematology/oncology	8 (15%)
Immune/inflammatory	8 (15%)
Peripheral vascular	3 (6%)
Other	8 (15%)
Number of hospitalizations in 2 years prior to COVID-19	
0	39 (75%)
1-3	11 (21%)
4-6	2 (4%)
COVID hospital length of stay in days, median (IQR)	4.5 (3-7)
Highest respiratory support	
Room air	6 (12%)
Nasal cannula/mask	31 (60%)
BiPAP/high flow nasal cannula	12 (23%)
Endotracheal intubation	3 (6%)
Lowest oxygen saturation	
≥ 90%	10 (19%)
80-89	38 (73%)
70-79	4 (8%)

*Note.* Sums of percents for comorbid conditions are >100% since most participants had >1 condition; other sums are >100% are due to rounding.

**Table 2:** Self-Reported Symptoms and Quality of Life (N=52)

	In hospital	In past 7 days (T1)	In past 7 days (T2)
Participants with ≥ 1 symptom	52 (100%)	50 (96%)	44 (85%)
Mean no. of symptoms per participant with symptoms (SD)	10.5 (5.0)	8.2 (4.1)	8.0 (4.0)
	Range 2-23	Range 1-18	Range 2-17
Lung	47 (90%)	36 (69%)	29 (56%)
Shortness of breath	41 (79%)	31 (60%)	25 (48%)
Difficulty breathing	36 (69%)	19 (37%)	14 (27%)
Dry cough	31 (60%)	22 (42%)	12 (23%)
Wet cough	14 (27%)	15 (29%)	9 (17%)
Neurological	46 (88%)	45 (87%)	36 (69%)
Foggy thinking	33 (63%)	28 (54%)	26 (50%)
Headaches	30 (58%)	21 (40%)	19 (37%)
Impaired taste	25 (48%)	16 (31%)	10 (19%)
Impaired smell	21 (40%)	13 (25%)	12 (23%)
Dizziness	19 (37%)	11 (21%)	10 (19%)
Memory difficulty	13 (25%)	25 (48%)	25 (48%)
Numbness/tingling in hands or feet	9 (17%)	11 (21%)	17 (33%)^†^
Nightmares	9 (17%)	4 (8%)	7 (13%)
Vision changes	4 (8%)	11 (21%)	9 (17%)
Hallucinations	4 (8%)	0	0
Musculoskeletal	40 (77%)	35 (67%)	27 (52%)
Muscle weakness	30 (58%)	31 (60%)	21 (40%)
Aches	30 (58%)	28 (54%)	24 (46%)
Gastrointestinal	36 (69%)	17 (33%)	17 (33%)
Lack of appetite	24 (46%)	5 (10%)	7 (13%)
Diarrhea	22 (42%)	12 (23%)	8 (15%)
Nausea	13 (25%)	4 (8%)	2 (4%)
Vomiting	5 (10%)	1 (2%)	1 (2%)
General	50 (96%)	47 (90%)	43 (83%)
Fatigue	47 (90%)	45 (87%)	40 (77%)
Fever	23 (44%)	1 (2%)	0
Pain	20 (38%)	23 (44%)	20 (38%)
Chills	17 (33%)	8 (15%)	7 (13%)
Runny nose	15 (29%)	13 (25%)	16 (31%)
Sore throat	9 (17%)	3 (6%)	5 (10%)
Skin discoloration	0	1 (2%)	1 (2%)
Other	3 (6%)	6 (12%)	6 (12%)
Symptoms have impacted ability to carry out normal routine	A lot	7 (13%)	3 (6%)
	Somewhat	28 (54%)	25 (48%)
	Not at all	17 (33%)	24 (46%)
Current quality of life compared to pre-COVID	Worse	36 (69%)	24 (46%)
	Same	14 (27%)	26 (50%)
	Better	2 (4%)	2 (4%)
Have returned to work since hospitalization (*n* = 35 working prior)		26 (74%)	30 (86%)

*Note:* Response options for symptoms in the past 7 days were: “a lot,” “somewhat,” and “not at all.” Columns titled “In past 7 days,” include participants who selected “a lot” or “somewhat” for that symptom. ^†^T2 numbness/tingling includes 9 with new report, 8 who also reported at T1; 3 with this symptom at T1 didn’t report it at T2. Change in proportions calculated using McNemar test; significant only for number of participants with ≥ 1 symptom (*p* = 0.014) and neurological (*p* = 0.013)

**Table 3:** Intercorrelations for Study Variables across Timepoints

	1	2	3	4	5	6	7	8 T1	8 T2	9 T2	10 T2	11 T2	12 T2	13 T2	14 T2	15 T2
1. Prior respiratory	-	−.04	.12	.03	.55**	.00	.21	.18	.17	.22	.27	.07	−.09	−.13	−.11	.10
2. Prior cardiac	-	-	.12	.04	.50**	−.14	−.20	−.23	−.03	−.25	−.18	−.11	.04	.06	.08	.01
3. Prior endocrine	-	-	-	.11	.35**	.14	.00	−.10	.15	.06	.19	−.08	−.02	−.02	.22	.03
4. Prior mental health	-	-	-	-	.36**	−.09	.21	.23	.03	.19	.13	.22	−.25	.15	−.17	−.01
5. No. comorbid	-	-	-	-	-	−.06	−.04	−.14	−.06	.03	.05	−.08	−.07	−.05	.00	−.02
6. Hospital LOS	-	-	-	-	-	-	.16	.03	.30*	.01	.16	−.07	.17	−.06	.10	.13
7. Hospital no. of symptoms	-	-	-	-	-	-	-	.50**	.41**	.22	.38**	.41**	−.54**	.06	−.25	.27*
8. No. symptoms T1	-	-	-	-	-	-	-	-	.46**	.24	.21	.53**	−.50**	.11	−.34*	.16
9. Depression T1	.31*	−.30*	.02	.35*	.10	−.11	.27	-	.38**	.80**	.63**	.58**	−.51**	.32*	−.61**	.54**
10. Anxiety T1	.14	−.12	−.12	.18	−.09	−.06	.22	-	.43**	.66**	.65**	.54**	−.42**	.40**	−.48**	.60**
11. Fatigue T1	.00	.01	−.30*	.24	−.04	−.22	.39**	-	.37**	.33**	.22	.78**	−.64**	.27	−.47**	.34*
12. Cognitive function T1	−.09	.11	.11	−.30*	−.03	.30*	−.60**	-	−.35*	−.27	−.29*	−.62**	.80**	−.16	.36**	−.31*
13. Sleep disturbance T1	.08	−.10	−.02	−.10	−.17	−.21	.16	-	.30*	.12	.28*	.49**	−.34*	.68**	−.32*	.33*
14. Satisfaction T1	−.06	.11	.02	−.20	.01	.06	−.25	-	−.47**	−.43**	−.23	−.55**	.45**	−.13	.58**	−.35*
15. IES-R T1	.21	−.04	−.05	.05	.00	.12	.27*	-	.40**	.35*	.42**	.48**	−.30*	.41**	−.47**	.68**

*Note.* **p < .01. *p < .05. Age only correlated significantly with prior cardiac condition (.43**) and fewer hospital symptoms (−.31*). IES-R = Impact of Events Scale-Revised.

**Table 4:** 

Table 4a.										
*Descriptive Statistics and Correlations for Study Variables within T1 (N=52)*										
Measure	Mean (SD)	Range	1	2	3	4	5	6	7	8
1. Depression (*n*=51)	45.9 (8.2)	38-67	–							
2. Anxiety (*n*=51)	46.4 (11.7)	35-85	.72**	–						
3. Fatigue	55.2 (10.2)	33-78	.52**	.49**	–					
4. Cognitive function	49.9 (10.8)	29-67	−.42**	−.42**	−.75**	–				
5. Sleep disturbance	51.4 (10.0)	31-78	.27	.43**	.35*	−.36**	–			
6. Satisfaction	43.6 (8.6)	26-66	−.49**	−.25	−.44**	.36**	−.12	–		
7. IES-R	13.5 (14.8)	0-67	.47**	.69**	.36**	−.36**	.53**	−.21	–	
8. No. symptoms	7.8 (4.3)	0-18	.41**	.30*	.63**	−.60**	.27	−.40**	.26	–

Table 4b.										
*Descriptive Statistics and Correlations for Study Variables within T2 (N=52)*										
Measure	Mean (SD)	Range	1	2	3	4	5	6	7	8
1. Depression (*n*=51)	44.8 (8.1)	38-67	–							
2. Anxiety	43.3 (10.1)	35-64	.79**	–						
3. Fatigue	51.6 (10.6)	33-71	.50**	.52**	–					
4. Cognitive function	52.5 (11.1)	33-67	−.43**	−.49**	−.74**	–				
5. Sleep disturbance	49.6 (9.1)	31-69	.24	.39**	.54**	−.33*	–			
6. Satisfaction	48.1 (9.1)	26-66	−.55**	−.44**	−.61**	.47**	−.44**	–		
7. IES-R	11.0 (14.4)	0-66	.59**	.59**	.50**	−.46**	.39**	−.59**	–	
8. No. symptoms	6.8 (4.7)	0-17	.44**	.63**	.62**	−.60**	.32*	−.45**	.58**	–

*Note. n* =52 unless otherwise indicated. Satisfaction = Satisfaction with Social Roles & Activities; IES-R = Impact of Events Scale-Revised. **p < .01. *p < .05.

**Table 5:** Resources and New Medical Equipment Used After Hospitalization (N=52)

	T1	T2
Any resource	49 (94%)	35 (67%)
Primary care provider	47 (90%)	22 (42%)
Specialist physician/clinic	23 (44%)	16 (31%)
Emergency department	7 (13%)	3 (6%)
Pharmacist	27 (52%)	11 (21%)
Rehabilitative services	11 (21%)	9 (17%)
Counseling, support group	7 (13%)	4 (8%)
Dietitian	4 (8%)	4 (8%)
Social services	1 (2%)	2 (4%)
Other resource	3 (6%)	2 (4%)
Any new medical equipment	22 (42%)	1 (2%)
Oxygen	14 (27%)	0
Lung expansion machine, incentive spirometer	4 (8%)	0
Mobility or transfer devices	4 (8%)	1 (2%)
Oximeter	2 (4%)	0
Glucometer	2 (4%)	0

*Note.* T1 resource includes resources reported from hospitalization to T1; T2 includes resources reported between T1 and T2. Rehabilitative services include physical therapist, occupational therapist, speech language pathologist. Medical equipment only reported once per participant, at time first reported since hospitalization (T1) or since last survey (T2). Oxygen includes 1 participant who previously required oxygen only at night but required continuous at T1; glucometer includes 1 with upgraded monitor and increased testing.

## Data Availability

The data is not available at this time due to continued analysis in process.
